# Individualized flow-controlled ventilation compared to best clinical practice pressure-controlled ventilation: a prospective randomized porcine study

**DOI:** 10.1186/s13054-020-03325-3

**Published:** 2020-11-25

**Authors:** Patrick Spraider, Judith Martini, Julia Abram, Gabriel Putzer, Bernhard Glodny, Tobias Hell, Tom Barnes, Dietmar Enk

**Affiliations:** 1 Department of Anaesthesia and Intensive Care Medicine, Medical University of Innsbruck, Innsbruck, Austria; 2 Department of Radiology, Medical University of Innsbruck, Innsbruck, Austria; 3 Department of Mathematics, Faculty of Mathematics, Computer Science and Physics, University of Innsbruck, Innsbruck, Austria; 4 University of Greenwich, London, UK; 5 Faculty of Medicine, University of Münster, Münster, Germany

**Keywords:** Respiration, artificial, Ventilator-induced lung injury, Tomography, X-ray computed, Pulmonary atelectasis, Stress mechanical, Respiratory mechanics

## Abstract

**Background:**

Flow-controlled ventilation is a novel ventilation method which allows to individualize ventilation according to dynamic lung mechanic limits based on direct tracheal pressure measurement at a stable constant gas flow during inspiration and expiration. The aim of this porcine study was to compare individualized flow-controlled ventilation (FCV) and current guideline-conform pressure-controlled ventilation (PCV) in long-term ventilation.

**Methods:**

Anesthetized pigs were ventilated with either FCV or PCV over a period of 10 h with a fixed FiO_2_ of 0.3. FCV settings were individualized by compliance-guided positive end-expiratory pressure (PEEP) and peak pressure (*P*_peak_) titration. Flow was adjusted to maintain normocapnia and the inspiration to expiration ratio (I:E ratio) was set at 1:1. PCV was performed with a PEEP of 5 cm H_2_O and *P*_peak_ was set to achieve a tidal volume (*V*_T_) of 7 ml/kg. The respiratory rate was adjusted to maintain normocapnia and the I:E ratio was set at 1:1.5. Repeated measurements during observation period were assessed by linear mixed-effects model.

**Results:**

In FCV (*n* = 6), respiratory minute volume was significantly reduced (6.0 vs 12.7, MD − 6.8 (− 8.2 to − 5.4) l/min; *p* < 0.001) as compared to PCV (*n* = 6). Oxygenation was improved in the FCV group (paO_2_ 119.8 vs 96.6, MD 23.2 (9.0 to 37.5) Torr; 15.97 vs 12.87, MD 3.10 (1.19 to 5.00) kPa; *p* = 0.010) and CO_2_ removal was more efficient (paCO_2_ 40.1 vs 44.9, MD − 4.7 (− 7.4 to − 2.0) Torr; 5.35 vs 5.98, MD − 0.63 (− 0.99 to − 0.27) kPa; *p* = 0.006). *P*_peak_ and driving pressure were comparable in both groups, whereas PEEP was significantly lower in FCV (*p* = 0.002). Computed tomography revealed a significant reduction in non-aerated lung tissue in individualized FCV (*p* = 0.026) and no significant difference in overdistended lung tissue, although a significantly higher *V*_T_ was applied (8.2 vs 7.6, MD 0.7 (0.2 to 1.2) ml/kg; *p* = 0.025).

**Conclusion:**

Our long-term ventilation study demonstrates the applicability of a compliance-guided individualization of FCV settings, which resulted in significantly improved gas exchange and lung tissue aeration without signs of overinflation as compared to best clinical practice PCV.

## Background

Ventilator-induced lung injury (VILI) is a serious complication in mechanically ventilated patients, significantly contributing to patient morbidity and mortality [1, 2]. Despite numerous efforts during the last decade to optimize standard ventilation methods in order to minimize the probability of VILI, e.g., by decreasing tidal volume (*V*_T_) and adjusting positive end-expiratory pressure (PEEP), the incidence of VILI is still high leading to pulmonary complications postoperatively as well as in ICU patients [1–3]. One inherent problem of artificial ventilation is the difficulty entailed in comprehensively determining individual lung mechanics from ventilation parameters and displayed measurements. In addition, setting the ventilator based on (predicted) body weight does not adequately address variations in lung mechanics [4].

Flow-controlled ventilation (FCV) is a ventilation mode in which flow is kept constant during both inspiration and expiration [5, 6]. This is a novelty in artificial ventilation. The resulting constant flow coupled with direct intratracheal pressure measurements allows much more precise analysis of individual lung mechanics than is possible in conventional ventilation modes, where flow varies over a wide range and intratracheal pressure is not directly accessible. In particular, FCV allows accurate determination of dynamic compliance and—based on this—precise adjustment of PEEP and peak pressure (*P*_peak_), thereby ensuring that ventilation occurs in the range between the (so-called) lower and upper inflection points of each patient’s pressure-volume curve.

In addition, it has been shown that the energy applied to the lung tissue is a significant factor contributing to the development of VILI [7]. Energy dissipation in lung tissue is related to flow. Therefore, ventilation at constant flow (which avoids the high flow peaks as in conventional ventilation) leads to minimization of applied and—more importantly—dissipated energy [5]. There is growing awareness that energy dissipation during the ventilation cycle is related to VILI and thus its reduction seems to be a key factor in developing more protective ventilation strategies [6].

The aim of this study was to evaluate the applicability of individualized FCV and to examine—for the first time—the effects of individualized FCV on respiratory and metabolic parameters as well as on lung aeration in comparison to evidence-based, best clinical practice pressure-controlled ventilation (PCV) during long-term ventilation in pigs.

## Methods

### Animal preparation

Experiments were performed in 12 domestic pigs weighing 35 to 45 kg. Animals were fasted overnight with free access to water. Intramuscular premedication was performed with azaperone (4 mg/kg) and atropine (0.5 mg) before transportation to the experiment facility.

Sedation was deepened with an intramuscular injection of ketamine (30 mg/kg). After being placed in supine position animals were intubated with an 8.0-mm internal diameter endotracheal tube (ETT) (Willy Rüsch GmbH, Kernen, Germany) followed by injection of propofol (2 mg/kg) and rocuronium (1 mg/kg) via an ear vein cannula. Anesthesia was maintained with a continuous infusion of propofol (6 to 8 mg/kg/h), remifentanil (0.2 to 0.3 μg/kg/h), and rocuronium (0.5 mg/kg/h). Following induction, baseline ventilation using volume-controlled ventilation (VCV) was initiated (Julian®; Dräger Medical, Lübeck, Germany) with an FiO_2_ of 0.3 and a *V*_T_ of 7 ml/kg body weight, a PEEP of 5 cm H_2_O and an inspiration-to-expiration ratio (I:E ratio) of 1:1.5. Respiratory rate (RR) was adjusted to maintain normocapnia (paCO_2_ 35 to 45 Torr; 4.7 to 6.0 kPa). Normovolemia was maintained by infusion of a balanced crystalloid solution (5 to 10 ml/kg/h Elomel iso®; Fresenius Kabi Austria GmbH, Graz, Austria).

This anesthetic regime has been proven to guarantee appropriate depth of anesthesia without hemodynamic disturbances [8].

Before starting invasive instrumentation 1.5 g cefuroxime was administered intravenously and repeated after 4 h to prevent septic complications. For invasive arterial pressure monitoring and arterial blood gas sampling an introducer sheath (5 F; Arrow, Reading, PA, USA) was advanced under ultrasound guidance via the femoral artery. A pulmonary artery catheter (7 F; Edwards Life Science, Irvine, CA, USA) was positioned via the right internal jugular vein after ultrasound-guided introducer sheath insertion (8.5 F; Arrow, Reading, PA, USA). A pig-tail catheter (8 F; Bard, Tempe, AZ, USA) was inserted into the bladder after ultrasound-guided puncture of the bladder for urine release and an esophageal probe (14 F; NutriVent, Sidam S.R.L., Mirandola, MO, Italy) positioned for monitoring of esophageal pressure (*P*_es_) as a surrogate parameter for pleural pressure.

### Experiment protocol

After instrumentation, the animal was allowed to stabilize for 15 min before baseline measurements were obtained and the protocol was started with pre-oxygenation followed by an apnea phase, where the tracheal tube was disconnected from the ventilator for 1 min.

Animals were randomized to FCV or PCV. In FCV animals the apnea phase was used to insert a 2.3-mm internal diameter endotracheal tube (Tritube®; Ventinova Medical B.V., Eindhoven, The Netherlands) into the standard ETT. Subsequently, ventilation was started with either FCV (Evone®; Ventinova Medical B.V., Eindhoven, The Netherlands) or PCV (Evita XL®; Dräger, Lübeck, Germany) with a fixed FiO_2_ of 0.3. PCV was performed with a PEEP of 5 cm H_2_O, *P*_peak_ set to achieve a *V*_T_ of 7 ml/kg and the RR adjusted to maintain normocapnia (paCO_2_ 35 to 45 Torr; 4.7 to 6.0 kPa). The I:E ratio was maintained at 1:1.5. FCV was performed with compliance-guided PEEP and *P*_peak_ settings (see below), and the flow was adjusted to maintain normocapnia. The I:E ratio was set at 1:1.

Measurement points were defined as T0 before commencement of the intervention period (baseline) with T1 to T14 at 0, 15, 30, 45, 60, 120, 180, 240, 300, 360, 420, 480, 540, and 600 min after initiating either FCV or PCV. Mechanical ventilation was performed for 10 h in supine position without any recruitment maneuvers. The study ended with a CT scan of the chest immediately after the intervention period.

### Individualization of flow-controlled ventilation (FCV)

**Fig. 1:**
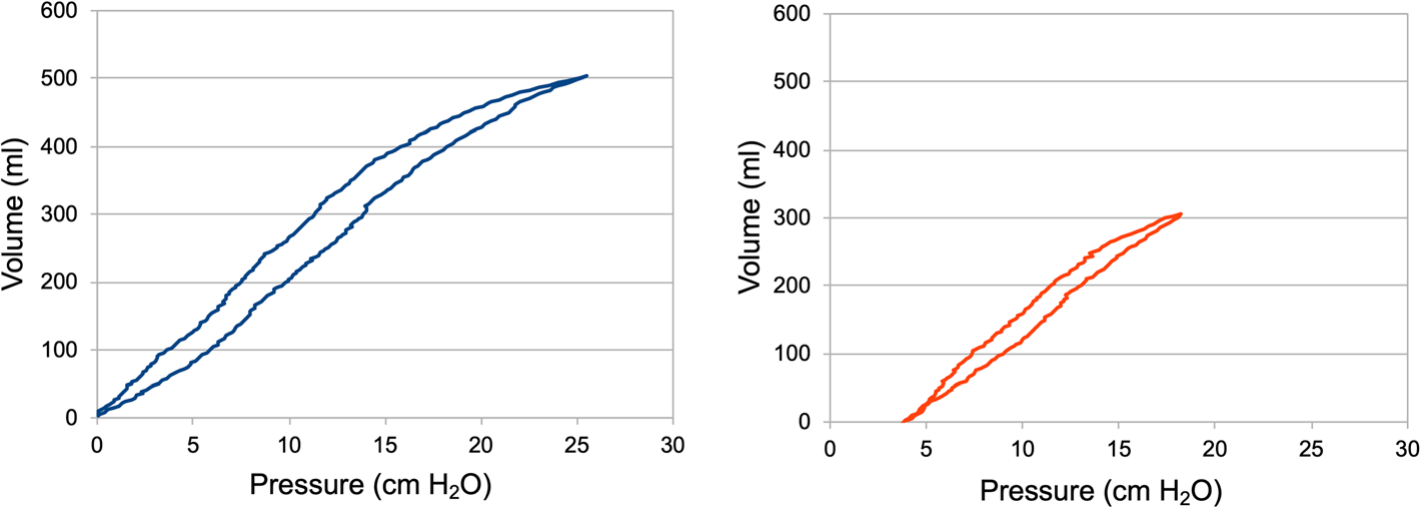
The pressure-volume loop (PV loop) obtained from intratracheal pressure measurement in a pilot animal. In the left graph, ventilation was performed without positive end-expiratory pressure (PEEP) and a peak pressure (*P*_peak_) of 25 cm H_2_O, showing a sigmoid shape of the PV loop. After compliance-guided pressure adjustment PEEP was set to 4 cm H_2_O and *P*_peak_ to 18 cm H_2_O, resulting in an almost linear relation between pressure and volume (right graph)

If the animal was randomized to FCV, ventilation was performed with the Evone® ventilator (Ventinova Medical B.V., Eindhoven, the Netherlands). Individualization of FCV by compliance-guided titration of PEEP and *P*_peak_ was performed as follows: first, the PEEP was stepwise increased or decreased while maintaining the same driving pressure until the highest *V*_T_ was reached. Subsequently, *P*_peak_ was increased stepwise as long as the *V*_T_ showed an—based on measured dynamic compliance—at least slightly overproportional rise (Fig. 1). Finally, the flow was set to maintain normocapnia at an I:E ratio of 1:1, which is best for minimizing dissipated energy [5, 6]. Thus, half of the flow roughly represented the respiratory minute volume (MV) for FCV.

### Respiratory and cardiovascular measurements

Respiratory and cardiovascular measurements were taken at T0 to T14 (defined above). MV, *V*_T_, and RR were recorded directly from the ventilator. Measured *P*_peak_ was documented as displayed for FCV as well as PCV, where an observed zero flow phase at *P*_peak_ indicated equilibrium between airway pressure and tracheal pressure. PEEP was additionally checked for intrinsic increments to rule out air trapping in PCV.

Arterial blood gas samples were obtained and paO_2_ and paCO_2_ measured (ABL800 Flex®; Radiometer, Brønshøj, Denmark).

Cardiovascular monitoring included heart rate (HR), mean arterial pressure (MAP), mean pulmonary arterial pressure (MPAP), and central venous pressure (CVP). Cardiac output (CO) and systemic and pulmonary vascular resistance (SVR, PVR) were measured by threefold injection of 10 ml of saline via the pulmonary arterial catheter. CO, SVR, and PVR indices were calculated using the predicted body surface area for pigs [9].

### Computed tomography and image postprocessing

To assess inspiratory and expiratory lung aeration two scans were performed with appropriate hold maneuvers lasting approximately 5 s to obtain images of the lung after 10 h of ventilation. The ventilation settings remained otherwise unchanged. All examinations were done with a Somatom Confidence® CT scanner (Siemens Healthineers, Erlangen, Germany). The settings were as follows: tube voltage 120 kV, tube current 600 mA (without exposure modulation), single collimation width 0.6 mm, slice thickness 0.75 mm, total collimation width 19.2 mm, table speed 57.6 mm, table feed per rotation 28.8 mm, spiral pitch 1.5, matrix 512 × 512, window center 50/-600, window width 350/1200 HU, convolution kernel I40f/3 and B70F, and a field of view 294 mm. For image processing an AW Server Workstation (AWS Version 3.2, Volume Viewer program; General Electric, Boston, MA, USA) was used. The lungs were segmented semi-automatically. Then, the total lung volume was determined automatically, as well as the lung volumes at different Hounsfield unit (HU) thresholds in 50 HU intervals. As described by Gattinoni et al. [10], non-aerated lung tissue was defined as absorption values between 100 and − 100 HU, poorly aerated lung tissue as values between − 101 and − 500 HU, normally aerated lung tissue as values between − 501 and − 900 HU, and airway as well as overdistended lung tissue as values between − 901 and − 1000 HU.

### Statistical analysis

A mathematician (TH) not involved in the study procedures performed the statistical analyses using R, version 3.5.3. Continuous data were presented as median (25th to 75th percentile) and categorical variables as frequencies (%). Effect size and precision were shown with estimated median differences between groups for continuous data and odds ratios (OR) for binary variables with 95% confidence intervals (CI). All statistical assessments were two-sided, and a significance level of 5% was used. The Wilcoxon rank sum test and Fisher’s exact test were applied to assess differences between the groups.

The progression of measurements from T0 to T14 was illustrated per group using the median course with corresponding 95% CI’s. Differences between groups were assessed with linear mixed-effects models with random intercepts for time points and subjects as well as group as fixed effects.

Differences in the Hounsfield unit (HU) distribution in non-aerated and normally aerated regions were assessed by applying the Wilcoxon rank sum test to the area under the curve.

## Results

The experiment protocol was completed in 12 pigs.

### Respiratory and cardiovascular measurements

Baseline characteristics were comparable in both groups except for a significantly higher MV in PCV animals due to a slightly (non-significantly) higher body weight (Table 1).

**Table 1:** Characteristics of laboratory animals before the start of the experiment

		Total (***n*** = 12)	FCV (***n*** = 6)	PCV (***n*** = 6)	MD with 95% CI^**a**^	***p*** value^**b**^
**Demographic data**						
Weight	kg	37.8 (33.8–43.3)	36.2 (32.9–38.7)	40.5 (36.6–44.8)	− 4.1 (− 11.6 to 2.3)	0.240
Size	m	1.08 (1.07–1.10)	1.08 (1.06–1.10)	1.08 (1.07–1.14)	−0.03 (− 0.09 to 0.03)	0.511
Gender	female	7/12 (58.3%)	4/6 (66.7%)	3/6 (50%)	0.53 (0.03 to 8.30)	1
**Monitoring data**						
HR	/min	89.0 (81.0–95.5)	91.0 (84.0–94.3)	85.0 (76.8–94.8)	3.9 (−8.0 to 20.0)	0.521
MAP	mm Hg	65.5 (63.0–72.0)	73.0 (66.5–75.75)	63.0 (63.0–65.3)	8.0 (−4.0 to 15.0)	0.106
MPAP	mm Hg	22.0 (20.0–23.3)	21.0 (20.3–24.8)	23.0 (20.8–23.0)	−0.5 (−4.0 to 8.0)	1
CVP	mm Hg	10.0 (8.0–13.0)	9.0 (8.0–10.0)	13.0 (10.8–13.0)	−3.0 (−6.0 to 6.0)	0.220
CI	l/min/m^2^	6.1 (5.7–6.6)	6.0 (5.6–6.4)	6.2 (5.9–7.5)	−0.4 (− 2.2 to 0.7)	0.485
PCWP	mmHg	11.0 (9.0–13.0)	10.0 (6.0–11.0)	12.5 (11.3–13.0)	−3.0 (−8.0 to 2.0)	0.195
*V*_T_	ml/kg	7.1 (7.1–7.5)	7.2 (7.1–7.7)	7.1 (7.0–7.3)	0.1 (−0.3 to 0.9)	0.191
RR	/min	35.0 (33.8–37.3)	34.0 (31.0–36.3)	36..0 (34.5–37.5)	−2.0 (−8.0 to 2.0)	0.332
MV	l/min	9.5 (9.0–11.6)	9.1 (8.9–9.4)	11.6 (10.1–11.8)	−2.4 (−4.3 to −0.1)	0.041^*^
*P*_peak_	cm H_2_O	19.5 (18.0–22.5)	18.5 (18.0–19.8)	23.0 (19.0–27.0)	−4.0 (−10.0 to 1.0)	0.195
Δ*P*	cm H_2_O	14.5 (13.0–17.5)	13.5 (13.0–14.8)	18.0 (14.0–22.0)	−4.0 (−10.0 to 1.0)	0.195
*P*_es_	cm H_2_O	12.0 (12.0–15.0)	12.0 (12.0–15.0)	12.5 (12.0–14.5)	0.0 (− 4.0 to 3.0)	0.924
paCO_2_	Torr	41.1 (39.3–43.3)	40.4 (39.0–42.8)	41.8 (40.0–42.9)	−0.9 (−4.6 to 2.5)	0.699
	kPa	5.47 (5.24–5.77)	5.38 (5.20–5.70)	5.57 (5.33–57.20)	−0.12 (− 0.61 to 0.33)	
paO_2_	Torr	120.5 (97.8–127.3)	113.5 (99.8–123.5)	123.0 (95.8–130.8)	−4.5 (−30.0 to 34.0)	0.818
	kPa	16.07 (13.03–16.97)	15.13 (13.30–16.47)	16.40 (12.77–17.43)	−0.60 (−4.00 to 4.53)	

*CI* cardiac index, *CVP* central venous pressure, *FCV* flow-controlled ventilation, *HR* heart rate, *MAP* mean arterial pressure, *MD* mean difference, *MPAP* mean pulmonary arterial pressure, *MV* respiratory minute volume, *paCO*_*2*_ arterial partial pressure of carbon dioxide, *paO*_*2*_ arterial partial pressure of oxygen, *PCV* pressure-controlled ventilation, *PCWP* pulmonary capillary wedge pressure, *P*_*es*_ esophageal pressure, *P*_*peak*_ peak pressure, *RR* respiratory rate, *V*_*T*_ tidal volume, *ΔP* driving pressure (difference between positive end-expiratory pressure and peak pressure);

Binary data are presented as no./total no. (%), continuous data as medians (25th to 75th percentile)

^a^Odds ratios for binary variables and estimated median difference for continuous variables

^b^Assessed by Fisher’s exact test for categorical variables and Wilcoxon rank sum test for continuous variables

**Fig. 2:**
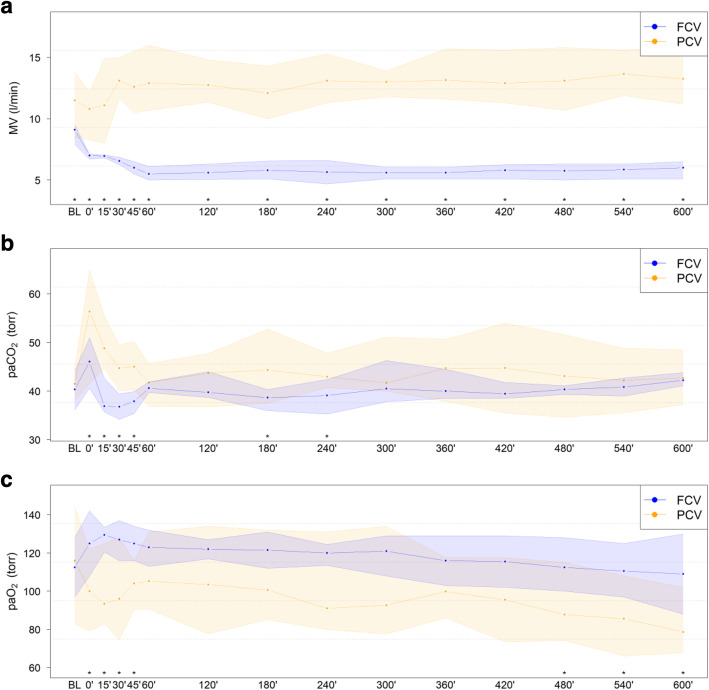
Course of parameters over 10-h ventilation with FCV or PCV. **a** Respiratory minute volume (MV). **b** Arterial partial pressure of carbon dioxide. **c** Arterial partial pressure of oxygen at a fixed 0.3 fraction of inspired oxygen

During the observation period of 10 h, *P*_peak_ was comparable in both groups, whereas PEEP was significantly lower in FCV (*p* = 0.002, Table 2). *V*_T_ was significantly higher in FCV than in PCV (8.2 vs 7.6, MD 0.7 (0.2 to 1.2) ml/kg; *p* = 0.025). RR in PCV was twice as high as in FCV (20.1 vs 41.5, MD − 21.3 (− 22.8 to − 19.9) /min; *p* < 0.001). MV was significantly lower in the FCV group (6.0 vs 12.7, MD − 6.8 (− 8.2 to − 5.4) l/min; *p* < 0.001, Fig. 2) than in the PCV group, as well as calculated mechanical power (5.8 vs 22.0, MD − 16.2 (− 21.1 to − 11.4) J/min; *p* < 0.001) [11]. FCV animals showed significantly improved oxygenation (paO_2_ 119.8 vs 96.6, MD 23.2 (9.0 to 37.5) Torr; 15.97 vs 12.87, MD 3.10 (1.19 to 5.00) kPa; *p* = 0.010, Fig. 2) as well as CO_2_ removal (paCO_2_ 40.1 vs 44.9, MD − 4.7 (− 7.4 to − 2.0) Torr; 5.35 vs 5.98, MD − 0.63 (− 0.99 to − 0.27) kPa; *p* = 0.006, Fig. 2) as compared to PCV animals.

**Table 2:** Course of parameters over 10 h with estimated differences between groups

		**FCV mean**^**a**^	**PCV mean**^**a**^	**MD with 95% CI**^**a**^	***p*** **value**^**a**^
HR	/min	84.0	79.6	4.4 (−5.0 to 13.8)	0.378
MAP	mmHg	70.1	65.1	5.0 (−0.3 to 10.4)	0.093
MPAP	mmHg	21.8	22.8	−1.0 (−4.8 to 2.8)	0.615
CVP	mmHg	9.5	12.1	−2.6 (−6.1 to 0.9)	0.177
CI	l/min/m^2^	5.4	5.7	−0.3 (−1.0 to 0.4)	0.410
PCWP	mmHg	9.5	12.6	−3.1 (−6.5 to 0.3)	0.108
PVRI	dyn s/cm^5^/m^2^	327.2	260.2	67.0 (20.9 to 113.1)	0.017^*^
SVRI	dyn s/cm^5^/m^2^	1646.1	1353.0	293.1 (51.4 to 534.7)	0.039^*^
*P*_es_	cm H_2_O	12.3	13.9	−1.6 (−3.7 to 0.5)	0.178
*V*_T_	ml/kg	8.2	7.6	0.7 (0.2 to 1.2)	0.025^*^
RR	/min	20.1	41.5	−21.3 (−22.8 to −19.9)	< 0.001^***^
*P*_peak_	cm H_2_O	16.3	17.5	−1.2 (−3.5 to 1.2)	0.350
ΔP	cm H_2_O	13.0	12.5	0.6 (−1.7 to 2.8)	0.638
MP	J/min	5.8	22.0	−16.2 (−21.1 to − 11.4)	< 0.001^***^
MV	l/min	6.0	12.7	−6.8 (−8.2 to −5.4)	< 0.001^***^
paCO_2_	Torr	40.1	44.9	−4.7 (−7.4 to −2.0)	0.006^**^
	kPa	5.35	5.98	−0.63 (−0.99 to −0.27)	
paO_2_	Torr	119.8	96.6	23.2 (9.0 to 37.5)	0.010^*^
	kPa	15.97	12.87	3.10 (1.19 to 5.00)	
PEEP		**FCV**^**b**^	**PCV**^**b**^		***p*** **value**^**c**^
5	cm H_2_O	1/6 (17%)	6/6 (100%)		0.002^**^
4	cm H_2_O	0/6 (0%)	0/6 (0%)		–
3	cm H_2_O	5/6 (83%)	0/6 (0%)		–

*CI* cardiac index, *CVP* central venous pressure, *FCV* flow-controlled ventilation, *HR* heart rate, *MAP* mean arterial pressure, *MD* mean difference, *MP* mechanical power, *MPAP* mean pulmonary arterial pressure, *MV* respiratory minute volume, *paCO*_*2*_ arterial partial pressure of carbon dioxide, *paO*_*2*_ arterial partial pressure of oxygen, *PCV* pressure-controlled ventilation, *PCWP* pulmonary capillary wedge pressure, *PEEP* positive end-expiratory pressure, *P*_*es*_ esophageal pressure, *P*_*peak*_ peak pressure, *PVRI* pulmonary vascular resistance index, *RR* respiratory rate, *SVRI* systemic vascular resistance index, *V*_*T*_ tidal volume, *ΔP* driving pressure (difference between positive end-expiratory pressure and peak pressure)

^a^Estimated mean and median difference with CI for continuous variables retrieved from linear mixed-effects model

^b^Binary data are presented as no./total no. (%)

^c^Assessed by Fisher’s exact test

Vascular resistance indices PVRI (327.2 vs 260.2, MD 67.0 (20.9 to 113.1) dyn s/cm^5^/m^2^; *p* = 0.017) and SVRI (1646.1 vs 1353.0, MD 293.1 (51.4 to 534.7) dyn s/cm^5^/m^2^; *p* = 0.039) were significantly higher in the FCV group although no effects on other hemodynamic parameters (HR, MAP, MPAP, CVP, and CI) were observed (Table 2).

### Computed tomography

**Fig. 3:**
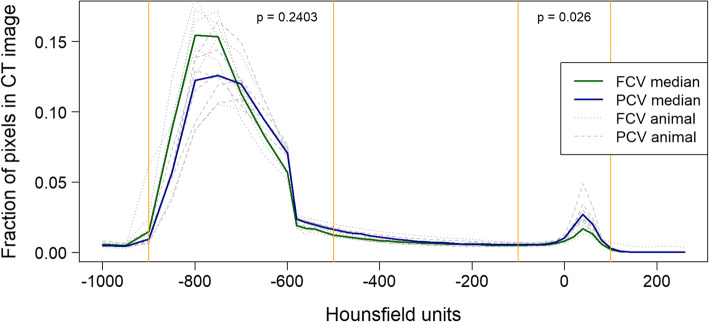
The Hounsfield unit (HU) distribution after 10 h of ventilation. Defining lung tissue aeration as non-aerated (HU 100 to − 100), poorly aerated (HU − 101 to − 500), normal aerated (HU − 501 to − 900), and overdistended (HU − 901 to − 1000) revealed no increase in overdistended, but a significant decrease in non-aerated lung tissue (*p* = 0.026)

Chest CT scans were obtained in inspiratory and expiratory hold. Classification of the lung tissue proportion at inspiration in overdistended (HU range of − 1000 to − 901), normally aerated (HU range of − 900 to − 501), poorly aerated (− 500 to − 101) and non-aerated (− 100 to 100) lung areas revealed a significant reduction in non-aerated lung tissue in FCV (*p* = 0.026, Fig. 3). Otherwise, there were no significant differences in overdistended, normally aerated and poorly aerated lung tissue in FCV as compared to PCV. Analysis of end-expiratory lung volume (EELV) revealed comparable results in both groups (FCV 545.9 vs PCV 604.6, MD − 19.6 (− 161.0 to 140.9) ml; *p* = 0.589).

In order to visualize the differences in ventilation mode, a cinematic CT scan of an FCV and PCV representative was obtained, which is provided as digital content (Additional file 1). RR was reduced to 10 per minute, otherwise the settings of the ventilator remained unchanged. The video clearly reveals the smooth and steady changes in lung volume during inspiration and expiration without any interruptions during the ventilation cycle in FCV. In contrast, when administering PCV rapid changes in lung volume can be noted, which are caused by decelerating flow during inspiration and expiration, resulting in pause phases without tissue movement.


**Additional file 1.**

## Discussion

The main finding of this study is the applicability of compliance-guided individualization of FCV settings. Tailored ventilator settings meet today’s ambitions of precision medicine and individual patient needs much better than do fixed numbers or thresholds for every patient.. We showed that compliance-guided pressure adjustment with FCV did not cause more regional overinflation of lung tissue when compared to evidence-based, low tidal volume PCV even though higher *V*_T_s were applied with FCV. Several studies report a significant improvement in ventilation efficiency and aeration of lung tissue when FCV is applied [12–16]; however, these studies did not use an individualized ventilation approach. Our study shows that normocapnia was maintained in individualized FCV despite a remarkable reduction of respiratory minute volume by 50% compared to PCV. Second, individualized FCV resulted in significantly improved oxygenation and a significant reduction of non-aerated lung tissue indicating an improvement of ventilation efficiency and lung aeration.

Several factors contributing to improved ventilation efficiency were found for FCV. First, in animals ventilated with FCV dead space ventilation was significantly reduced by increasing *V*_T_. The finding of increased *V*_T_ may be counterintuitive in terms of lung-protective ventilation at first sight since studies have clearly shown that higher tidal volumes may be associated with a negative outcome in ARDS [17]. However, the primary idea of FCV is to physically reduce dissipated energy applied to lung tissue as much as possible. The only way to minimize dissipated energy is to keep gas flow constant during the entire ventilation cycle and at the lowest possible level [5, 6]. With minimized gas flow, ventilation must be as efficient as possible; therefore, tidal volume was increased within lung mechanic limits to reduce dead space ventilation but simultaneously decrease the risk of atelectasis and/or overdistension (Fig. 1). This process is aided by the viscoelastic properties of the lung tissue—it has more time to “creep” and relieve stress in FCV. In fact, the combination of direct intratracheal pressure measurement and a constant flow allows for the first time to measure dynamic compliance during ventilation and pressure settings were adjusted accordingly. The novelty in individualized FCV is that tidal volume is naturally strongly related to individual lung compliance as a result of the individualization process, thereby representing the ventilation of the available aerated lung tissue. This can lead to a higher *V*_T_ in lung healthy individuals (as shown in this study) but would also result in decreased *V*_T_ if the compliance of an injured lung is reduced.

In addition, improved lung aeration with less atelectasis was observed in FCV animals, even though a lower PEEP level was established than with PCV. These findings agree with those of previous studies, where a recruiting effect due to a linearized expiratory airway pressure decline was supposed for FCV [12, 13]. In fact, controlling expiratory flow mimics physiological effects provided by the glottis (acting as a dynamic resistor to the egress of gas) and the diaphragm (slowing down expiratory flow by controlled muscle relaxation). As already mentioned above, the observed higher *V*_T_ when administering FCV did not increase the proportion of overdistended lung tissue. This fact underlines previous findings in non-individualized FCV showing a more even ventilation pattern [14–16]. The observed better lung aeration and more homogeneous gas distribution improve ventilation efficiency, not only by increasing gas exchange surface and diffusion capacity, but also by reducing intrapulmonary shunt fraction and improving the ratio between ventilation and perfusion. However, further studies are needed to investigate the exact shunt fraction and ventilation/perfusion ratio in FCV.

The results of a trial in healthy piglets suggest that the energy delivered by the ventilator to the lung tissue contributes to VILI [7]. However, established calculations for mechanical power applied to the lung tissue consider only the inspiratory effort since expiration is presumed to be a passively occurring process. In earlier work on the concept of FCV, we already hypothesized that energy dissipated in the lung tissue during both inspiration and expiration is an important contributor to VILI [5, 6]. In mechanical ventilation, energy is necessarily dissipated because of resistive work that needs to be performed in order to overcome airway and tissue resistance during inspiration as well as expiration. Part of the applied energy is stored during inspiration (by expanding the (visco)elastic lung tissue and chest), can be partly recovered during expiration (by elastic recoil), and leaves the lung tissue again (during the egress of respiratory gas). By contrast, the dissipated energy (which represents the unavoidable “loss” of energy of any working mechanical system) remains in the lung tissue. In order to minimize dissipated energy, a constant flow of respiratory gas at the lowest level is most favorable [5]. Decelerating flow in PCV during inspiration and expiration results in two spikes where energy is dissipated. Contrarily, constant flow in FCV lacks any energy spikes delivered to and dissipated in the lung tissue [6]. Therefore, individualized FCV is performed not only with the greatest efficiency with respect to lung mechanics at the lowest flow guaranteeing normoventilation, but also with the lowest possible energy input to lung tissue.

Moreover, dynamic stress and strain, both undisputed contributors to VILI [17–20], are reduced in FCV due to steady, slow changes in pressure and smooth increase or decrease in lung volume during the whole ventilation cycle at an optimally low RR (see digital content: Additional file 1).

Our study has several limitations. First, the measured technical dead space of the Evone® ventilator is 25 ml lower than that of the EvitaXL® (50 ml vs. 75 ml), contributing to a higher MV in the PCV group. Moreover, the RR required to achieve normocapnia was significantly higher with PCV. However, correction of MV with consideration of technical and RR-related dead space ventilation effects still yields a significant MV reduction in FCV animals (5.0 vs 9.6 l/min). Taking into account the significantly higher baseline MV of 2.4 l/min in the PCV group due to a slightly higher body weight (Table 1), a considerably reduced MV still results in the FCV animals (approx. 5.0 vs. 7.1 l/min). This aligns our findings of improved ventilation efficiency with previous findings [12].

Second, PEEP in the FCV group was adjusted by means of measurement of dynamic lung mechanics. In the PCV group, however, PEEP was set at 5 cm H_2_O according to the recommendations of the ARDS Network [17] for low tidal volume ventilation in healthy lungs. We acknowledge, this may not represent a truly “fair fight” between FCV and PCV; however, a PEEP level of 5 cm H_2_O was used in a previous preclinical study investigating the effects of FCV versus VCV making our results very roughly comparable to this trial [12]. It may be argued that oxygenation could be improved by applying a slightly higher PEEP level in the PCV group; on the other hand, we observed improved oxygenation in the FCV group despite a significantly *lower* PEEP level. Finally, the aim of the current study was to compare ventilation based on evidence-based fixed numbers with settings based on an individualized approach.

Although more recent studies suggest using personalized ventilator settings based on electrical impedance tomography measurements and repeated CT scans [4], this approach was not feasible in our study due to a lack of resources and does not reflect clinical routine nor was it possible to perform PEEP titration guided by transpulmonary pressure due to anatomical issues related to the measurement of esophageal pressure (*P*_es_) as a surrogate parameter for pleural pressure (Table 1). The location of the esophagus is far more dorsal in pigs than in humans and therefore *P*_es_ reflects only pleural pressure at the very dependent parts of the lungs and would have led to unacceptably high PEEP titration in order to keep transpulmonary pressure positive. Besides the fixed PEEP level in PCV, no recruitment maneuvers were performed either with PCV or FCV. Recruited lung tissue might have led to redistribution of the fixed tidal volume in PCV and thus a more homogeneous distribution of transpulmonary pressure through interdependence properties and, after an increase of lung compliance, a reduction of driving pressure. In the absence of recruitment maneuvers, it has to be stated that ventilator settings based on inspiratory pressure-volume curves may not provide enough information to guarantee safe mechanical settings.

Third, it is not possible to directly quantify atelectasis in the Hounsfield unit (HU) distribution, because atelectatic tissue lies in the same HU range as blood vessels and soft and fat tissue (− 100 to 100). Therefore, technically it is not possible to distinguish between atelectasis and non-alveolar lung tissue. However, since the amount of non-alveolar lung tissue inside the thoracic cavity should be roughly equal in healthy pigs, we hypothesize that the significant differences seen in the CT scan presentation are attributable to atelectasis. Additionally, CT images were taken during a hold maneuvers representing a static CT acquisition of a dynamic process; this fact is even more important for FCV than for PCV as in FCV normally any pause during ventilation is absent.

Finally, this study investigated lung healthy pigs with a comparable low compliance compared to humans. Thus, accepted VILI indicators such as tidal volume, driving pressure, and mechanical power are not fully transferable to humans, especially not under different pathophysiological conditions such as ARDS.

## Conclusion

This porcine long-term ventilation study demonstrates the applicability of compliance-guided individualization of FCV settings. Additionally, compared to low tidal volume PCV, more efficient gas exchange with improved oxygenation, more efficient CO_2_ elimination, and more homogeneous gas distribution without signs of overinflation in FCV were confirmed. Our results for individualized FCV and the underlying concept for reducing dissipated energy (according to accepted thermodynamic considerations in the technical field) and minimizing stress and strain are suggestive of a more lung-protective ventilation strategy than is the current best clinical practice PCV. Further studies and clinical trials are consequently needed to determine and investigate long-term effects of individualized FCV under different pathophysiological conditions.

## Data Availability

The datasets used and analyzed during the current study are available from the corresponding author on reasonable request.

## Abbreviations

ARDS: Acute respiratory distress syndrome
CI: Cardiac index
CI: Confidence interval
CO: Cardiac output
CT: Computed tomography
CVP: Central venous pressure
EELV: End-expiratory lung volume
ETT: Endotracheal tube
FCV: Flow-controlled ventilation
FiO_2_: Fraction of inspired oxygen
HR: Heart rate
HU: Hounsfield unit
ICU: Intensive care unit
I:E ratio: Inspiration to expiration ratio
MAP: Mean arterial pressure
MD: Median difference
MPAP: Mean pulmonary arterial pressure
MV: Respiratory minute volume
OR: Odds ratio
paCO_2_: Arterial partial pressure of carbon dioxide
paO_2_: Arterial partial pressure of oxygen
PCV: Pressure-controlled ventilation
PCWP: Pulmonary capillary wedge pressure
PEEP: Positive end-expiratory pressure
*P*_es_: Esophageal pressure
*P*_peak_: Peak pressure
PVR(I): Pulmonary vascular resistance (index)
RR: Respiratory rate
SVR(I): Systemic vascular resistance (index)
VCV: Volume-controlled ventilation
VILI: Ventilator-induced lung injury
*V*_T_: Tidal volume
ΔP: Driving pressure
